# Effect of Processing Steps on the Mechanical Properties and Surface Appearance of 6063 Aluminium Extruded Products

**DOI:** 10.3390/ma7064224

**Published:** 2014-05-30

**Authors:** Juan Asensio-Lozano, Beatriz Suárez-Peña, George F. Vander Voort

**Affiliations:** 1 Materials Science and Metallurgical Engineering Department, School of Mines, University of Oviedo, Center Campus, Oviedo, Asturias 33004, Spain; 2 Materials Science and Metallurgical Engineering Department, Polytechnic School of Engineering, University of Oviedo, Campus of Gijón, Gijón, Asturias 33203, Spain; E-Mail: bsuarez@uniovi.es; 3 Struers Inc., 2887 N. Southern Hills Drive, Wadsworth, IL 60083-9293, USA; E-Mail: georgevandervoort@yahoo.com

**Keywords:** casting and forming technologies, alloy design, 6063 aluminium alloys, quantitative metallographic characterization, mechanical characterization, light optical microscopy (LOM), scanning electron microscopy (SEM), streak formation

## Abstract

6063 aluminum anodized extrusions may exhibit a common surface defect known as streaking, characterized by the formation of narrow bands with a surface gloss different from the surrounding material. The origin of this banding lies in the differential surface topography produced after etching during the anodizing stage, shown to be connected to certain microstructural characteristics. The present study has attempted to determine the origin of these defects and measure the mechanical properties in these zones, properties which were either barely acceptable or did not meet the specification’s requirements. Quantitative metallography and mechanical testing, both tensile and microhardness, were used for materials assessment at the different steps of the process of manufacturing 6063 anodized extrusions. The results of this research show that nonequilibrium solidification rates during billet casting could lead to the formation of coarse eutectic Mg_2_Si particles which have a deleterious effect on both mechanical properties and surface appearance in the anodized condition. However, differences in the size and density of the coarse Mg_2_Si particles have been found to exist in the streak profile compared to the surrounding zones. The study revealed the importance of these particles in explaining the origin of the marginal or sub-marginal properties and anodizing surface defects found.

## 1. Introduction

The 6063 Al-Mg-Si alloy is widely used in the manufacture of shaped aluminum profiles produced by extrusion. The billets are obtained via the direct chill (DC) casting method. During DC casting, liquid metal is poured into a water-cooled mold (primary cooling). While the skin of the ingot becomes solid, the inside still remains semi-solid/liquid. Further cooling of the ingot bulk is achieved by quenching the solid shell directly using water jets (secondary cooling) [1]. The cooling rate (d*T*/d*t*) varies from 1 K/s in the center of the billet to around 20 K/s in its surface zone [2]. The phases formed in the different zones of the billet depend on their chemical composition and cooling rate. Furthermore, the extrusion formability as well as the mechanical and surface extruded properties depends on the chemical composition and degree of homogeneity of the billet [3,4]. Therefore, 6063 aluminum alloys are usually subjected to a homogenization heat treatment prior to extrusion [5]. This heat treatment causes a reduction in microsegregation of solute atoms and partial solution of Mg and Si from pre-existing Mg_2_Si particles. Fine magnesium silicide precipitates (β-Mg_2_Si) are responsible for the potential strength of the alloy [6,7,8]. After heat treatment, the homogenized bars are cut into slugs, preheated and then forced into an extrusion press. The extrusions may be hyper-quenched and aged, depending on the extrusion thickness [9]. The profile is then subjected to etching prior to anodizing. During etching, etching pits are produced due to the different reaction rates between the existing particles (Mg_2_Si and AlFeSi) and the aluminum matrix. Finally, the profiles are subjected to an anodizing process, during which an oxide layer is formed on top of the aluminum surface. Any surface imperfections at the metal oxide interface will increase the diffuse part of the reflected light and thus result in streak defects [10].

Etching pits, grain boundary grooves and grain etching steps are the most common surface defects created during etching process. The presence of surface imperfections does not always result in streaking. Streak defects are only observed when there is an uneven distribution of surface imperfections. Billet quality, including billet microstructure, chemical composition and billet contamination may influence or directly result in the inhomogeneous distribution of the surface microstructure. Imperfect extrusion processes can generate mixing of alloys with different chemical compositions resulting in inhomogeneous microstructure yielding regions of different intermetallic particles [11]. Streaking may also occur when the ram approaches the extrudate too closely at the end of the stroke. The surface of the original billet can flow into the core of the extrusion and stretch to the surface of the extrudate, ultimately resulting in streaking [12]. Also non-uniform metal flow and heat generation due to the friction and plastic deformation during extrusion may result in an inhomogeneous surface microstructure such as grain size, grain orientation and precipitates which has been reported as a source for streak defects that can be observed after anodizing [13].

This paper reports the effect of several process steps during the fabrication of anodized 6063 extrusions, analyzing the mechanical properties and surface appearance in extruded sections. The study considers all the processing steps in the process chain. The analyzed casting process variables comprise the chemical content in Mg, Si and Fe. As regards the homogenization process, the studied variables were the microstructural evolution and mechanical properties, obtained by tensile testing, after treatment of the billet. Further down the process line, extrusion and anodizing were analyzed in terms of morphology, size and distribution of Mg_2_Si and AlFeSi particles, and the influence of these particles on surface appearance and mechanical properties, in particular microhardness.

## 2. Results and Discussion

**Figure 1:**
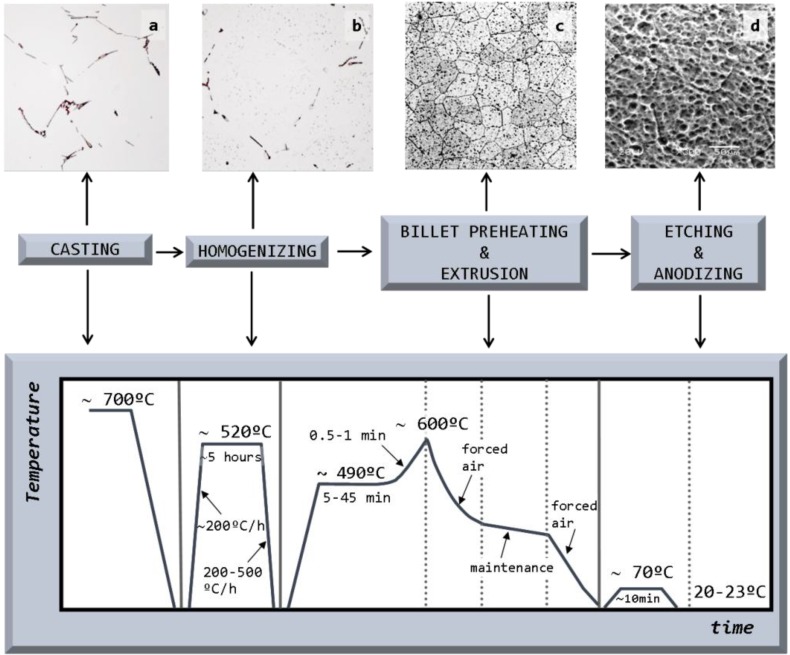
The different stages of the manufacturing process of extrusions from semi-continuous cast billets. Correlation with its corresponding microstructure: (**a**) after casting; (**b**) in the as-heat treated condition; (**c**) after extrusion; and (**d**) after anodizing. Note: Scale bars for magnification have not been included here since these micrographs are the same as those in Figure 3a,b and Figure 7a,c.

Figure 1 outlines the different stages of the manufacturing process, showing the microstructural evolution in terms of the different phases present in the samples. After casting, the samples were analyzed in the heat-treated condition following extrusion and anodizing.

### 2.1. Billet Quality

**Figure 2:**
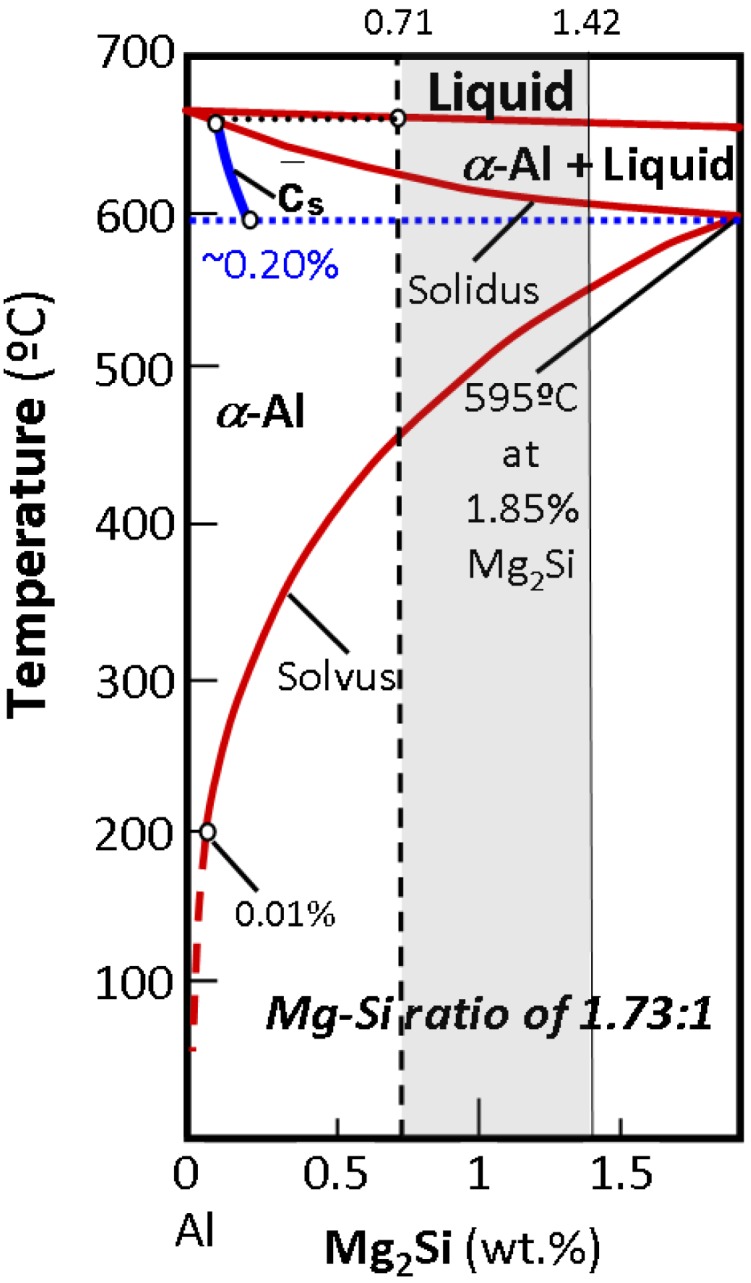
Pseudo-binary Al-Mg_2_Si phase diagram for a Mg:Si weight ratio of 1.73:1. The displacement of the average composition of the solid under nonequilibrium conditions corresponding to industrial solidification practices is shown.

In the ternary Al-Mg-Si system, Mg_2_Si particles are formed as a result of a ternary peritectic reaction. However, in the pseudo-binary Al-Mg_2_Si system, obtained from a vertical section of the Al-Mg-Si ternary system at a fixed Mg:Si atomic ratio of 2:1, equivalent to 1.73:1 when the same ratio is expressed as a quotient of weights (Figure 2), these Mg_2_Si particles could be formed as the equilibrium reaction products of a eutectic reaction [14]:

*L*_E_(13.9 wt%) → α-Al (1.85 wt%) + Mg_2_Si (*T*_E_ = 595 °C) (1)

where the concentrations are expressed in weight percent of Mg_2_Si.

Given that the Mg limits the solubility of Mg_2_Si in α-Al, while the Si has no effect on it, a weight ratio of Mg:Si ≤ 1.73 is sought when designing industrial alloys in order to ensure the maximum amount of this compound in solution [15]. The compositional limits for Mg and Si in 6063 alloys range between 0.45–0.9 wt% and 0.2–0.6 wt%, respectively [14]. Assuming that all the Mg is present in the Mg_2_Si form, the amount of Si needed to form Mg_2_Si precipitates for the compositional range of Mg in 6063 alloys is given by:

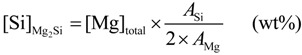
(2)
where [Si]_Mg_2_Si_ is the amount of Si expressed in wt% present in the Mg_2_Si compound; [Mg]_total_ corresponds to Mg interval values for 6063 alloys; and *A*_Si_ and *A*_Mg_ are the atomic weights of Si and Mg, respectively.

The Mg_2_Si concentration, [Mg_2_Si], can be calculated according to:

[Mg_2_Si] = [Si]_Mg_2_Si_ + [Mg]_total_ (wt%) (3)

In addition, for the chosen Mg values in the range of compositions found in 6063 alloys, the composition in Mg_2_Si will thus vary between 0.71 and 1.42 wt% (Figure 2).

If solidification takes place under equilibrium conditions, the only constituents present in the billet will be solid solution α-Al and Mg_2_Si precipitates, occurring in the solid state and nucleated at triple points and grain boundaries. The weight fraction of this equilibrium solid state precipitate, *f*_w_(Mg_2_Si), can be estimated at 200 °C instead of at RT, given that solid state diffusion can be considered to be negligible at temperatures equal or below 200 °C. The maximum solubility of Mg_2_Si at this temperature has been assumed to be ~0.1 wt%-Mg_2_Si.


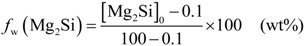
(4)
where [Mg_2_Si]_0_ is the initial weight percent composition in Mg_2_Si. The calculated weight fractions, *f*_w_, of precipitates for the above compositions, 0.71 and 1.42 wt%-Mg_2_Si, are 0.611 and 1.321 wt%, respectively.

The improvement in mechanical properties in these alloys is obtained by precipitation hardening, consisting of fine, uniform precipitation of Mg_2_Si obtained by ageing from the supersaturated state of α-Al. The sequence of precipitation is as follows [16]:

Supersaturated Al solid solution → Clusters of Si and Mg atoms → GP zones → Intermediate precipitate β″-Mg_2_Si → Intermediate precipitate βʹ-Mg_2_Si → Equilibrium phase β-Mg_2_Si

#### 2.1.1. Billet Casting

Hsu *et al.* [17] have shown that in most 6xxx Al alloys, solidification takes place via the formation of primary Al followed by secondary eutectic and peritectic reactions leading to the formation of small amounts of intermetallic particles in interdendritic regions. At a typical cooling rate for DC casting, a cubic AlFeSi phase has been observed to form mainly via two reactions: (i) equilibrium peritectic reactions L + Al_13_Fe_4_ → α-Al + AlFeSi; and (ii) non-equilibrium eutectic reaction L → α-Al + AlFeSi. Tanihata *et al.* [18] also found that, for industrial cooling rates (~5 K/s) and when the Fe:Si ratio is lower than 1, monoclinic β-AlFeSi particles are formed preferentially to cubic α_c_-AlFeSi particles suggesting a typical atomic ratio of Al:Fe:Si for particles within grains of 5:1:1 [18,19].

In the studied alloy, the composition in iron is 0.19 wt% (Table 1). According to the technical literature, this iron will combine to form Al_5_FeSi intermetallics prior to the formation of Mg_2_Si compounds. The compositional design of the alloy should thus allow for an excess of Si in order to ensure that a sufficient amount of this element is readily available to combine with the total Mg, thus maximizing the fraction of Mg_2_Si precipitates that can be formed. The amount of the Si necessary for the formation of AlFeSi intermetallic compounds can be estimated from the chemical composition of the billet:

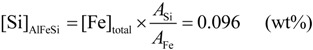
(5)
where [Si]_AlFeSi_ is the amount of Si in wt% in the AlFeSi intermetallic; [Fe]_total_ is the amount of Fe in wt% in the alloy, *i e.*, 0.19; and *A*_Si_ and *A*_Fe_ are the atomic weights of Si and Fe, respectively.

Hence, the total available Si for further combination will be 0.354 wt%. The Mg:Si weight ratio for the alloy under study (Table 1) is 1:1. The Si needed for Mg_2_Si formation can be calculated according to Equation (2), the result being: [Si]_Mg_2_Si_ = 0.26 wt%. Our results indicate that there will be enough Si to form Mg_2_Si and that an excess of silicon, Si_xs_, will still remain in the solid solution of α-Al after combination, *i.e*., [Si]_xs_ = 0.094 wt%.

**Table 1:** Chemical composition* of commercial billet used in the study.

Alloy	Chemical composition (wt%)							
6063	Si	Fe	Cu	Mn	Mg	Zn	Ti	Al
	0.45	0.19	0.02	0.04	0.45	0.03	0.02	Balanced

* Trace levels of Ag, Zr, Sb, Co, V, Be, B, Ca, *etc.* were detected but are not reported.

The composition of the alloy expressed as wt%-Mg_2_Si, when all the Mg combines with Si after AlFeSi formation, can thus be calculated from Equation (3), yielding [Mg_2_Si] = 0.71 wt%. The value thus obtained coincides with the lower interval value of the compositional range for commercial 6063 alloys (Figure 2).

**Figure 3:**
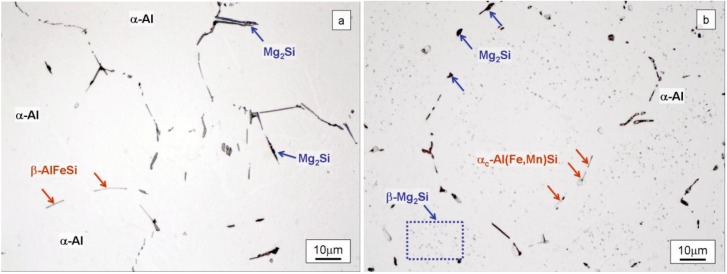
Light optical microscopy (LOM) microstructure of the billet in the intermediate zone after etching with 0.5% HF aqueous solution (**a**) in the as-cast billet it is possible to observe β-AlFeSi plate-like particles (light grey) and Mg_2_Si coarse particles originate from non equilibrium solidification both located at grain boundary and (**b**) after homogenization treatment β-AlFeSi needles transforms into α-AlFeSi discontinuous fragments in grain boundary (light grey) and Mg_2_Si particles transform into round particles (black).

Figure 3a shows the LOM microstructure of the alloy in the as-cast condition. A matrix of α-Al grains and a network of coarse Mg_2_Si particles distributed along grain edges and interdendritic spaces can be distinguished in this figure. The marked hypoeutectic nature of the alloy justifies the morphology found for these particles which have evolved from the S+L stage, as the volume reserved for these compounds to grow is limited to confined areas in the plane of observation, such as triple points and primary Al-α dendrite contours. After etching in a 0.5% HF aqueous solution, coarse Mg_2_Si particles become distinguishable in the LOM because they develop dark tones, whereas Fe-rich intermetallic particles show up in light grey tones.

The presence of coarse Mg_2_Si particles (Figure 3a) is eutectic in origin. High solidification rates during DC casting actually impede the homogenization of the solid grains, resulting in solidification by “layers” in the growing dendrites, which are progressively enriched in the Mg_2_Si solute. This phenomenon, known as microsegregation or “coring”, is more pronounced the higher the rate of solidification, which means a lower time for the solid to be homogenized via diffusion [20]. Establishing the average composition of the heterogeneous solid at each temperature *c*_*s*_, (Figure 2), its value at the eutectic temperature, *T*_E_ = 595 °C, may be found to be lower than that of the starting composition [Mg_2_Si]_0_. In this circumstance, liquid with a eutectic composition may exist, thereby giving rise to the reaction: *L*_E_→ α-Al + Mg_2_Si. The eutectic Mg_2_Si phase can be observed as coarse particles in LOM (Figure 3a). After these particles have formed, an excess of Mg_2_Si solute, [Mg_2_Si]_xs_, may still be found to exist in solid solution of α-Al. This will allow the formation of fine precipitates of β-Mg_2_Si in the solid state, responsible for structural hardening, as indicated by the solvus curve in the equilibrium pseudo-binary diagram (Figure 2).

Previous calculations showed that the composition of the analyzed billet yields a value for [Mg_2_Si] of 0.71 wt%, which is below the maximum solubility limit of Mg_2_Si in α-Al under equilibrium conditions, 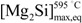, *i.e*., 1.85 wt% (Figure 2). The aforementioned solidification strategy entails a new maximum Mg_2_Si solubility limit, 
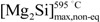
, for α-Al under non-equilibrium conditions. This value has to be derived from the average composition of the solid at the eutectic temperature and because of the presence of liquid phase at this temperature: 
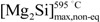
 < 1.85 wt% [21]. The solubility limit in the specific DC non-equilibrium condition, 
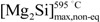
, is calculated as follows:

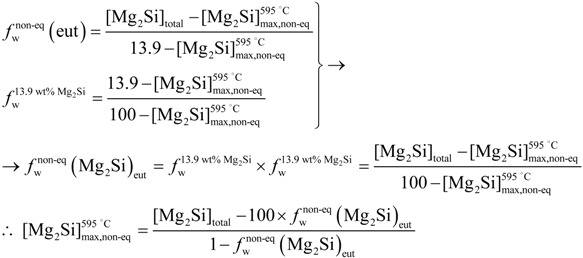
(6)
where [Mg_2_Si]_total_ is the total available Mg_2_Si concentration, *i.e*., 0.71 wt%, and 
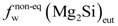
 is the eutectic Mg_2_Si weight fraction.

**Figure 4:**
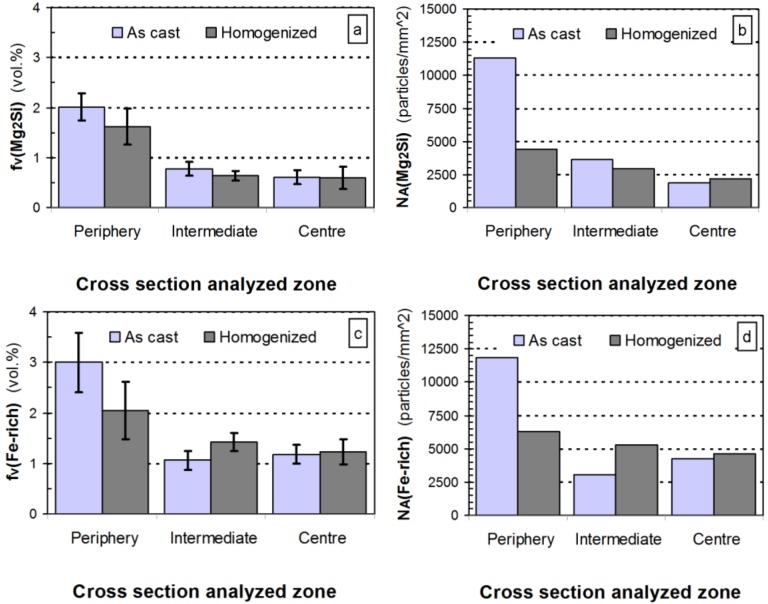
Quantitative determinations conducted in selected zones of the cross section of the billet. Samples were analyzed in both the as-cast state and after homogenizing. The determinations for Mg_2_Si particles are: (**a**) volume fraction, *f*_v_; and (**b**) particle density in the area, *N*_A_. And the determinations for AlFeSi intermetallics were also; (**c**) volume fraction, *f*_v_; and (**d**) areal particle density, *N*_A_. Error bars represent the 95% confidence limit of the determinations.

Quantitative metallographic techniques only allow the estimation of coarse eutectic Mg_2_Si volume fraction precipitates, 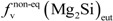, (Figure 4a). Assuming the specific weights for Mg_2_Si and for the 6063 alloy to be 1.99 [22] and 2.69 g × cm^−3^, respectively [14], it is possible to calculate the eutectic Mg_2_Si weight fraction, 
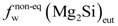
. As for the calculations, we took into consideration the quantitative metallographic determinations of the most representative areas in the billet, *i.e*., the intermediate zone and the central zone. The mean value of the eutectic Mg_2_Si volume fraction, 
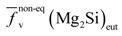
, is 0.69 vol% (Figure 4a), its corresponding calculated weight percent being calculated according to:

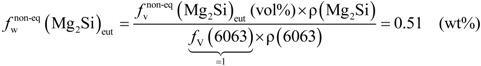
(7)

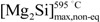
 can hence be operated from the final expression in Equation (6):

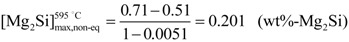
(8)

As for the above calculation, the non-equilibrium solubility limit of α-Al, 
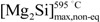
, will be set at ~0.20 wt%-Mg_2_Si (Figure 2). The weight fraction of fine precipitates that originated in the solid state, *f*_w_(β-Mg_2_Si), will thus be:

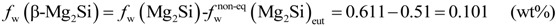
(9)

The calculations indicate a small amount of the finer β-Mg_2_Si precipitates, thus anticipating the scant contribution to the total strength that they can provide.

Observation of the results obtained by quantitative metallography to the billet at different locations (Figure 4) reveals a typical macrosegregation defective state that occurs in the semi-continuous DC casting of 6xxx billets, leading to uncontrolled redistribution of alloying elements during solidification. This manufacturing defect is responsible for large-scale composition inhomogeneities, commonly referred to as inverse segregation [1]. At the casting temperature in the solid state, the billet microstructure is characterized by weak α-Al grain boundaries. Via the action of metallostatic pressure, highly segregated liquid in the center vein of the billet is forced to migrate centrifugally towards the outer part through the weak α-Al grain boundaries at casting temperatures. This process leads to localized enrichment of the solute at a microscopic level near the external surfaces [23]. The result is caused by the movement of this interdendritic liquid towards the outer parts of the billet, occasionally leading to exudation when the liquid reaches the billet surface. The latter explains the higher densities of Mg_2_Si and AlFeSi particles observed on the periphery of the billet (Figure 4b,d). Macrosegregation in DC-cast ingots produces non-uniform mechanical properties [24]. These differences in microstructure between the surface and the bulk of billet can also produce a variation in the surface microstructure of the extrudate. Therefore, after etching the surface of the final extruded product could present a heterogeneous distribution of etching pits prior to anodizing, eventually leading to streak formation [13].

### 2.2. Homogenization Heat Treatment

#### 2.2.1. Microstructural Aspects

Observation of the microstructure in the as-heat treated samples (Figure 3b) shows the presence of α_c_-Al(Fe,Mn)Si particles in light grey located at the grain boundaries of α-Al grains, with rounded edges at the tips, as well as coarse particles of Mg_2_Si likewise located at the grain boundaries and fine β-Mg_2_Si precipitates forming a fine dispersion inside the grains.

Throughout the heating process employed during industrial treatment and the subsequent controlled cooling, monoclinic acicular β-AlFeSi phase transforms into cubic rounded α_c_-Al(Fe,Mn)Si, an excess of Si from this transformation being transferred to the Al matrix. This β→α conversion is strongly dependent on the chemical composition of the alloy [25]. The presence of elements such as Mn and Si favors the change [26]. After treatment, the Fe-based intermetallic compounds are located at the grain boundaries of α-Al, but do not form a continuous network (Figure 3b).

The amount of Si needed for the formation of α_c_-Al(Fe,Mn)Si precipitates obtained after heat treatment, [Si]_Al(Fe,Mn)Si_, differs from that calculated in the previous Section (5) for β-AlFeSi. Calculations are made based on both the chemical composition of the billet and the stoichiometry of α_c_-Al(Fe,Mn)Si particles [19]. Onurlu *et al.* [27] have shown that if the Mn content exceeds the value of 0.014 wt%, the cubic phase is stable, the most common formulation for this precipitate being given by α_c_-Al_12_(Fe,Mn)_3_Si:

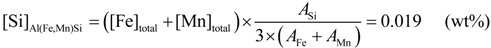
(10)
where *A*_Mn_ is the atomic weight percentage of Mn, [Mn]_total_ being the weight percent of manganese in the alloy, *i.e*., 0.04.

The excess of Si after heat treatment, [Si]_xs_, will be given by:

[Si]_xs_ = [Si]_total_ − [Si]_Al(Fe,Mn)Si_ − [Si]_Mg_2_Si_ = 0.45 − 0.019 − 0.26 = 0.171 (wt%) (11)

Following heat treatment, the amount of Si in solid solution of aluminium is ~1.8 times higher than in the as-cast condition.

The results obtained after applying quantitative metallographic techniques to quantify the particles (Figure 4) shows that inverse segregation persists after heat treatment. In fact, the highest values of the volume fractions, *f*_v_, correspond to the periphery of the billet, decreasing sharply towards the center (Figure 4a,c), following a similar trend to that observed in the as-cast state.

As a result of the heat treatment, both types of precipitate tend to show a decrease in their volume fraction. While this decrease occurs significantly in the peripheral region of the billets, it is barely noticeable in the intermediate and central zones. As regards eutectic Mg_2_Si precipitates in particular, the homogenization treatment leads to partial disaggregation of the eutectic system and thus of Mg_2_Si precipitates [14]. Figure 4a shows that both the duration and temperature in the treatment were insufficient to achieve total solubilization of the eutectic precipitates. Slight disaggregation of these precipitates is observed only in the zone subjected to a higher heat intake, *i.e*., the periphery. Furthermore, during heat treatment, the elongated particles tend to break up into smaller ones in order to reduce their surface area per unit volume, thus minimizing their surface energy. This will lead to an increase in the density of these particles, *N*_A_, following heat treatment. If the breaking up of these elongated particles is accompanied by a decrease in their volume fraction, *f*_v_, due to partial solubilization, their density may be lower than that of the as-cast condition (Figure 4b,d).

**Figure 5:**
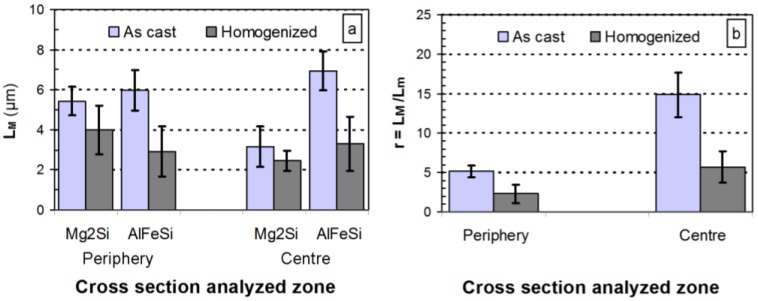
Particle morphology determinations assessed in selected zones of the billet cross section both in the as-cast state and after homogenizing. (**a**) Maximum Feret diameter, *L*_M_, for Mg_2_Si and AlFeSi particles; and (**b**) shape ratio *r* = *L*_M_/*L*_m_ in AlFeSi intermetallics. Error bars represent the 95% confidence limit of the determinations.

Quantitative metallography also allows the determination of the morphological changes that occur in the precipitates, in terms of both size and geometry, as a result of the industrial treatment applied (Figure 5). Due to the elongated nature of interdendritic particles, the quantitative metallographic parameter which best characterizes their size is the maximum particle length, *L*_M_, which has to be measured on individual particles. Figure 5a shows the decrease in maximum particle length after heat treatment for both types of precipitates in the periphery and center of the billet. Moreover, the higher this parameter is in the as-cast condition, the greater the variation in *L*_M_ after treatment. Figure 5b shows the ratio of the maximum to the minimum lengths in Fe-rich particles before and after treatment, both in the periphery and the center of the billet under study. The results show that the shape after treatment is less equiaxed (*r* ≈ 6) in the central zone than in the periphery (*r* ≈ 2). The result also highlights the low heat input applied to the center of the billet, as previously indicated in the provisional quantitative metallographic findings.

#### 2.2.2. Mechanical Characterization

**Figure 6:**
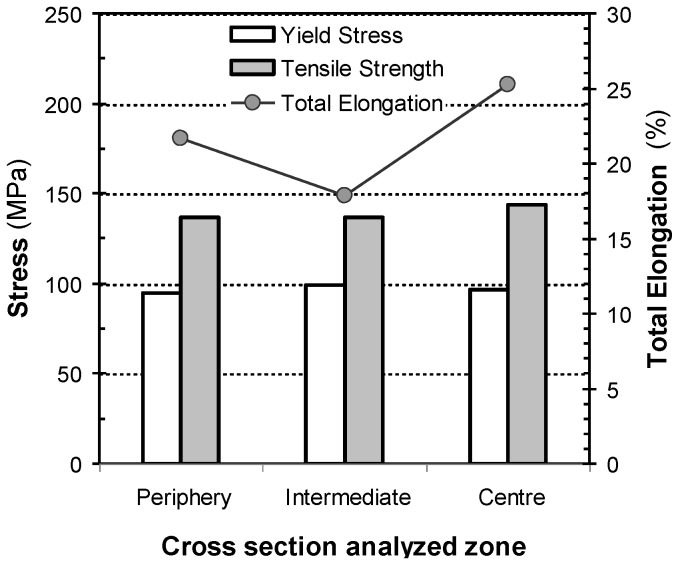
Tensile test results of the as-homogenized billet at three locations: periphery, intermediate and center. Tensile specimens machined parallel to the billet length.

The microstructural heterogeneities observed in the different areas of the billet after heat treatment are reflected in the heterogeneous values of the tensile parameters obtained after the testing of machined specimens extracted from the periphery, the intermediate zone and the center of the billet in the as-treated condition. Tensile test results show homogeneous values in terms of yield stress and tensile strength in all analyzed areas (Figure 6).

### 2.3. Billet Preheating and Extrusion

The metal during the extrusion process undergoes severe deformation to adapt to the new shapes following a streamline course of flow determined in accordance with the geometrical conditions of the die [28]. The influence of deformation history and related recrystallization effects results in an inhomogeneous microstructure and different zones can thus be observed. A dead metal zone forms in the boundary region at the front of the billet where friction between the billet, container and die inhibits significant deformation [29]. The material undergoes significant deformation which predominantly occurs by shearing and compression leading to the concept of redundant deformation, which is more pronounced the closer the unit volume of material in the billet, is to the walls of the extrusion container. In the center of the billet the metal entering the deformation zone changes it shape by compression without shear [30].

#### 2.3.1. Microstructural Aspects

The extrusion process commences with preheating of the slug to a temperature of around 490 °C, the goal being to dissolve the Mg and Si from the fine solid state β-Mg_2_Si precipitates. Solutionizing of precipitates is highly dependent on the particle surface:volume ratio. The finer the precipitates, the greater their surface:volume ratio and the faster they dissolve [31]. The increase in the size or non-uniform distribution of coarse Mg_2_Si particles decreases the extrusion speed and the increase in solid solution of Mg and Si in the billet can cause a decrease in the maximum extrusion speed [32].

**Figure 7:**
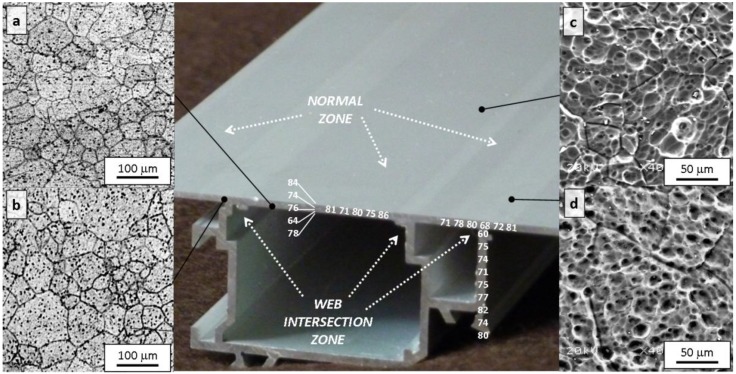
The photograph in the center shows the outer seen part of the anodized profile flange. It depicts the presence of bright streaks alternating with dull bands. Vickers microhardness values corresponding to the cross section are also indicated (white lettering). Upper and lower LOM micrographs to the left of central picture correspond to the cross section according to: (**a**) normal zone (N) and (**b**) web intersection zone (WI). Upper and lower SEM micrographs to the right of the central photograph correspond to the profile flange surface, according to; (**c**) normal zone (N); and (**d**) web intersection zone (WI).

Figure 7 shows a photograph of the center of the extruded profile. The extrusion cross-section microstructures observed under LOM in both the normal zone (N zone) and the web intersection zone (WI zone) are shown in Figure 7a,b. Mg_2_Si precipitates are revealed as dark dots. Table 2 shows the quantitative metallographic results on samples analysed in the extrusion cross-section.

The recrystallized grain size, *L*, was found to be similar in both areas, *i.e*., 52.37 µm in the N zone and 52.31 µm in the WI zone. Differences were detected between the two areas, however, in the density of coarse Mg_2_Si particles, *N*_A_. The value of *N*_A_ for the Mg_2_Si particles in the WI zone is 11.55 percent lower than that in the N zone. After extrusion, the cross-section areas where the flange and webs meet, namely WI zones (Figure 7), are initially subjected to lower deformation compared to contiguous zones. This results in less fragmented Mg_2_Si particles after extrusion deformation. Thus, the value of *N*_A_ for Mg_2_Si particles in the WI zone will be lower than that in N zone. In conclusion, the WI zone in the profile cross-section has less Mg_2_Si particles, but these are larger in size, *d*_p_, compared to those found in the N zone.

**Table 2:** Microstructural and mechanical results in the cross section of the extruded billet.

Zone	α-Al Grain size				Mg_2_Si particles		HV (200g)		*σ*_TS_ (MPa)	*σ*_y_ (MPa)
	*L* (µm)	CL-95% (µm)	*g_e_* (ASTM)	CL-95% (ASTM)	*N*_A_ (part./mm^2^)	*d*_p_ (µm)	*HV* (Kg/mm^2^)	CL-95% (Kg/mm^2^)		
N	52.37	2.32	5.2	0.13	5546	2.29	76.95	±4.70	181.52	135.12
WI	52.31	2.77	5.2	0.15	4972	2.65	74.80	±3.27	175.92	130.73

#### 2.3.2. Mechanical Characterization

The Vickers microhardness test results shown in Figure 7 correspond to the average values, *HV*, given in Table 2. The average microhardness value in the N zone, 76.90 kg/mm^2^, is slightly higher than that found in the WI zone, 74.80 kg/mm^2^. The technical literature reveals that the hardness value of alloys is a function which depends on grain size and on the density and size of their precipitates. High hardness values are mainly obtained with small α-Al grain sizes and high densities of submicroscopic β-Mg_2_Si precipitates [33,34]. Given that strengthening via coarse precipitation likewise influences hardness values, though to a lesser extent, the particle density for this coarse fraction should also be taken into account. The observed differences in hardness for the N zones and WI zones cannot be attributed to a difference in α-Al grain size, as the *L* value obtained in both areas was found to be similar (Table 2).

However, the observed differences in hardness may be justified on one side, by the higher values of *N*_A_ and the smaller size, *d*_p_, obtained for the coarse Mg_2_Si fraction measured in the N zone. And on the other side, by the greater amount of strain-induced β-Mg_2_Si precipitates, expected in the N zone due to the somewhat higher unitary deformation in extrusion compared to that in the WI zone.

A reasonably accurate correlation has been found between hardness and tensile strength for many metals and alloys. Petty [35] conducted a detailed study of the relationships between Vickers hardness and tensile strength and yield stress, proposing the following equations:

σ_TS_ = 0.189 × *HV* −1.38 (ton/inch^2^) (12)

and,

σ_y 0.2%_ = 0.148 × *HV* −1.59 (ton/inch^2^) (13)

The results in Table 2 are presented SI units being the following equivalence used for conversion purposes: 1 ton/inch^2^ = 13.7895 MPa. The tensile strength values ranged from ~176 to ~182 MPa and yield stress values ranged from ~131 to ~135 MPa. If the above values are compared with those expected for the 6063-T5 alloy [14], equal to 186 MPa for the tensile strength and 145 MPa for the yield stress, it can be seen that the tensile mechanical properties are lower than expected. Marchive [36], derived a correlation between tensile strength and chemical composition for 6xxx alloys. Marchive suggested that for alloys containing 0.6–1 wt% of Mg_2_Si and 0.05–0.2 wt% of Si_xs_ the expected tensile strength can be determined by:

σ_TS_ = −55.5 + 300×[Mg_2_Si] _total_ + 872×[Si] _xs_-700×([Mg_2_Si] _total_ × [Si]_ xs_) (MPa) (14)

The calculations indicate that the optimum strength in this alloy should be 221.63 MPa, which is approximately 18% higher than the highest value obtained in the as-extruded sample referred to the former theoretical value (Table 2).This is attributable to the low transformation of the total Mg_2_Si solute in β-Mg_2_Si precipitates.

### 2.4. Etching and Anodizing

The photograph of the extruded profile in Figure 7 shows bright streaks alternating with dull bands. These streak defects are only visible in the anodized extruded surface above the web zone (flange). Banding defects of this kind can be associated with the two previously studied zones in the cross-section, *i.e*., the WI and N zones. The SEM micrographs in Figure 7c,d show the surface profile irregularities after anodizing. Grain boundary grooves and etch pits of variable depth and width, can be observed in this figure. Differences in average grain size can be disregarded as a possible origin for streak formation as a result of quantitative metallographic determinations (Table 2). Pits occur during etching due to the different reaction rates between the Mg_2_Si and AlFeSi particles compared to the α-Al matrix. The Fe-rich intermetallic compounds have a higher electrochemical potential than the α-Al matrix, becoming the cathode of the local galvanic cell and thus causing the dissolving of the α-Al matrix around these particles. AlFeSi are consequently detached from the surface once buoyancy forces allow this phenomenon to occur. The size of the etching pits is always larger than the size of the intermetallic particles, and it has a far-reaching influence in the optical surface appearance [13]. Larger amounts of the Fe-rich intermetallic particles can engender more etching pits after etching, hence leading to a lower surface gloss [37]. In contrast, Mg_2_Si particles act as anodes, dissolving away [10]. As a result, pits are produced on the surface of the extrusion after etching with size directly related to the original size of the particles. Hence Mg_2_Si precipitates can make a significant contribution to achieving a matte finish due to a high density of small pits created by the particles superimposed on the larger pits caused by Fe-rich intermetallic particles” [13].

Due to the transparent nature of the anodized film formed on top, incident light will be reflected to a different extent depending on size and depth of, pits. Since etching pits are related to the density and size of the pre-existing particles, streaks will be visible if the values measured for these parameters are different in contiguous bands (Figure 7a,b). In effect, the WI zone presented both, the lowest *N*_A_ value alongside the highest *d*_p_ value of the Mg_2_Si particles, leading to the widest and deepest pits formed after etching (Table 2). The N zone exhibits a somewhat higher *N*_A_ value (closer pits), but a relatively small Mg_2_Si particle diameter (shallow pits). It seems reasonable, therefore, that the differences detected between both contiguous areas could be responsible for the appearance of bands with different reflectivity, which are cause for rejection of the profiles.

## 3. Experimental Method and Materials

The chemical composition of the commercial billet is given in Table 1. The billet was manufactured via a DC semi-continuous process in which molten aluminum enters the top of a water-cooled mold, while a solid ingot is withdrawn from below. The molten aluminum begins to solidify as it comes into contact with the mold wall, forming a thin solid layer around a liquid melt. As the ingot is withdrawn from the bottom of the mold, water jets impinge directly on the solid surface, allowing completing solidification in the rest of the ingot.

The study commenced by sectioning a 20-mm-thick sample from a billet of 6000 mm in length and 178 mm in diameter. The remainder of the billet was introduced in an industrial furnace, where it was subjected to a homogenization treatment. This treatment consisted in heating to temperatures of around 520 °C for 5 h [7,38]. Subsequently, the billet was transferred to a cooling chamber where it was cooled first in still air and then under forced air. Once the process was completed, samples were extracted for subsequent microscopic observation and mechanical characterization by tensile testing. Metallographic analysis was conducted on the billet samples both in the as-cast and homogenized condition. Samples were taken from the periphery *i.e*., the material located around 200 μm below the billet surface, intermediate zone and center of the billet. Observations were made using light optical microscopy (LOM) techniques. The polished surfaces were etched in an aqueous solution of 0.5% HF [39,40,41]. Quantification of the volume fraction, areal density, morphology and size of the particles present in the samples, was performed using the quantitative metallographic techniques [42,43,44]. The volume fraction, *f*_v_, and areal density of the particles, *N*_A_, were determined by the manual point counting method [45] At least twenty-five fields were analyzed in each of the three examined zones in order to obtain a reasonable degree of accuracy [46]. To conduct the morphological characterization of the particles, their maximum (*L*_M_) and minimum (*L*_m_) lengths were measured, and the ratio between these values, *r* = *L*_M_/*L*_m_, was subsequently calculated. When r-ratio approaches a value of ~10, particles are assumed to have an acicular morphology, whereas when *r*-ratio reaches the value of 1, the particle is said to have rounded morphology [19,47]. Observations were performed on a Nikon Epiphot metallographic bench connected to a Kappa ImageBase semi-automatic image analyzer.

Subsequently, suitable amounts of the homogenized billet were cut into slugs and used for full-scale extrusion. The slugs were preheated to a temperature of around 490 °C prior to deformation. Experimental extrusions were cooled first in forced air, aged in a reverberatory chamber and finally cooled to room temperature (RT) in forced air (Figure 1). Mechanical characterization by Vickers microhardness tests was then performed on the cross-section. Metallographic characterization of the cross-section of the extruded profile was likewise conducted. For this purpose, samples were prepared by mechanical methods (grinding and polishing), followed by etching in an aqueous solution of 0.5% HF for LOM observation. An estimate of the grain size of the α-Al phase was obtained by means of the linear intercept method. At least 900 grains were analyzed in order to obtain a reasonable degree of accuracy [46]. Furthermore, the average areal density, *N*_A_, and the mean size of the Mg_2_Si particles, *d*_p_, present in the extrusion were also determined.

Before the extrusions were anodized, an alkaline etching step in a NaOH solution was applied. The anodizing stage follows, which was carried out in sulfuric acid at RT over a period of 40 min. Cross-sectional samples were cut from the extrusion for subsequent metallographic analysis. Two characteristics zones in the profile section were analyzed: the zones of intersection of the webs with the flange, called web intersections zones (WI zones), and the rest of the flange, called normal zones (N zones). Metallographic observation of the samples was performed using a JEOL-5600 SEM without any mechanical or chemical preparation due to the transparent nature of the anodized layer.

### 3.1. Mechanical Characterization

We chose to carry out tensile tests and Vickers hardness tests for the mechanical characterization of the samples. After heat treatment, cylindrical specimens were extracted from the periphery, intermediate zone and center of the billet slice. These samples were machined in the longitudinal direction of the billet. Tensile tests were subsequently carried out in accordance with ASTM B557-10 [48] on an INSTRON universal testing machine (model 5582), equipped with a 10 kN load cell, at a constant crosshead speed of 10 mm/min, with a 50-mm-length calibrated extensometer attached to the specimen. The following mechanical properties were determined in all cases: the yield strength at 0.2%, σ_y,0.2%_, the tensile strength, σ_TS_, and the total elongation, *A*_T_. A series of Vickers microhardness tests were also carried out on the WI and N areas in the cross-section of the extruded profile. The applied load was 200 g.

## 4. Conclusions

This paper provides a link between the mechanical properties, surface appearance and processing steps of an industrial 6063 extruded profile. Through the study of the microstructural features such as the morphology, size and distribution of Mg_2_Si and AlFeSi intermetallics, it has been made possible to correlate their influence on the surface appearance and mechanical properties in the final product.

The surface appearance and mechanical behavior in the extrusion depend on the resulting microstructure after casting, in particular on the nature of the Mg_2_Si phase. Typical cooling rates employed in industrial solidification lead to the formation of a high volume fraction of coarse Mg_2_Si precipitates. Commercial homogenizing treatment has proven to be insufficient to cause significant disaggregation of the coarse eutectic Mg_2_Si component. These eutectic precipitates may cause deterioration of the mechanical properties of the extruded profile, leading to surface defects. After anodizing, streaks of different brightnesses were observed on the surface flange of the extruded billet. These are the result of the dissimilar reflection of incident light beams on the surface of contiguous regions. These regions correspond in the cross-section with web intersection zones and adjacent flange areas referred to as normal zones. Quantitative metallographic techniques helped to determine the α-Al grain size, the results revealing no differences between web intersection and normal zones. Consequently, and notwithstanding the findings reported other research papers, in the present study grain size can be disregarded as a possible source of streak formation.

Volume fraction and particle areal density determinations have also enabled a better understanding of non-equilibrium solidification in a commercial 6063 alloy, in particular of the formation of coarse-Mg_2_Si precipitates. These precipitates seem to play a role in the different contrast observed on the surface of anodized extrusions. The results in both analyzed zones have shown that if there is a significant difference in areal density and in the size of fragmented Mg_2_Si precipitates, changes in the topography on the surface of the anodized profiles could appear and streaking may be observed.
